# The Progenetix oncogenomic resource in 2021

**DOI:** 10.1093/database/baab043

**Published:** 2021-07-17

**Authors:** Qingyao Huang, Paula Carrio-Cordo, Bo Gao, Rahel Paloots, Michael Baudis

**Affiliations:** Department of Molecular Life Sciences, University of Zurich, Winterthurerstrasse 190, Zurich 8057, Switzerland; Swiss Institute of Bioinformatics, Winterthurerstrasse 190, Zurich 8057, Switzerland; Department of Molecular Life Sciences, University of Zurich, Winterthurerstrasse 190, Zurich 8057, Switzerland; Swiss Institute of Bioinformatics, Winterthurerstrasse 190, Zurich 8057, Switzerland; Department of Molecular Life Sciences, University of Zurich, Winterthurerstrasse 190, Zurich 8057, Switzerland; Swiss Institute of Bioinformatics, Winterthurerstrasse 190, Zurich 8057, Switzerland; Department of Molecular Life Sciences, University of Zurich, Winterthurerstrasse 190, Zurich 8057, Switzerland; Swiss Institute of Bioinformatics, Winterthurerstrasse 190, Zurich 8057, Switzerland; Department of Molecular Life Sciences, University of Zurich, Winterthurerstrasse 190, Zurich 8057, Switzerland; Swiss Institute of Bioinformatics, Winterthurerstrasse 190, Zurich 8057, Switzerland

## Abstract

In cancer, copy number aberrations (CNAs) represent a type of nearly ubiquitous and frequently extensive structural genome variations. To disentangle the molecular mechanisms underlying tumorigenesis as well as identify and characterize molecular subtypes, the comparative and meta-analysis of large genomic variant collections can be of immense importance. Over the last decades, cancer genomic profiling projects have resulted in a large amount of somatic genome variation profiles, however segregated in a multitude of individual studies and datasets. The Progenetix project, initiated in 2001, curates individual cancer CNA profiles and associated metadata from published oncogenomic studies and data repositories with the aim to empower integrative analyses spanning all different cancer biologies. During the last few years, the fields of genomics and cancer research have seen significant advancement in terms of molecular genetics technology, disease concepts, data standard harmonization as well as data availability, in an increasingly structured and systematic manner. For the Progenetix resource, continuous data integration, curation and maintenance have resulted in the most comprehensive representation of cancer genome CNA profiling data with 138 663 (including 115 357 tumor) copy number variation (CNV) profiles. In this article, we report a 4.5-fold increase in sample number since 2013, improvements in data quality, ontology representation with a CNV landscape summary over 51 distinctive National Cancer Institute Thesaurus cancer terms as well as updates in database schemas, and data access including new web front-end and programmatic data access.

**Database URL**: progenetix.org

## Introduction

Copy number aberrations (CNAs) are present in the majority of cancer types and exert functional impact in cancer development (1, 2). As understanding cancer biologies remains one of the main challenges in contemporary medical and life sciences, the number of studies addressing genomic alterations in malignant diseases continues to grow. Progenetix is a publicly accessible cancer genome data resource (*progenetix.org*) that aims to provide a comprehensive representation of genomic variation profiles in cancer, through providing sample-specific CNA profiles and associated metadata as well as services related to data annotation, meta-analysis and visualization. Originally established in 2001 with a focus on data from chromosomal Comparative Genomic Hybridization (CGH) studies (3), the resource has progressively incorporated data from hundreds of publications reporting on genome profiling experiments based on molecular cytogenetics (CGH, genomic arrays) and sequencing (whole-genome or whole-exome sequencing—WGS or WES). Since the last publication dedicated to the Progenetix resource in 2014 (4), changes in content and features of the data repository and its online environment have vastly expanded its scope and utility to the cancer genomics community. For data content, additions include the complete incorporation of the previously separate arrayMap data collection (5, 6) and of datasets from external resources and projects such as The Cancer Genome Atlas (TCGA; (7, 8)) or cBioPortal (9), as well as the recurrent collection and re-processing of array-based data from National Center for Biotechnology Information (NCBI)’s Gene Expression Omnibus (GEO) or European Molecular Biology Laboratory-European Bioinformatics Institute (EMBL-EBI)’s ArrayExpress (10, 11). Additionally, data content updates have followed the previous methodology of publication-based data extraction where feasible. Beyond the data expansion, a tight integration with projects of the Global Alliance for Genomics and Health (GA4GH (12)) and ELIXIR—such as serving for implementation-driven development of the Beacon application programming interface (API) (13)—has led to an extension of the resource’s features as well as adoption and promotion of emerging open data standards.

Here we present the latest updates on data content, structuring, standardization, access and other modifications made to the Progenetix resource.

## Data expansion and new features

### Genomic profiling data

Over the last two decades, thousands of cancer genome studies have used the GEO (14) for deposition of data from array-based experiments. Data from GEO contribute a substantial fraction of the genomic screening data in the Progenetix collection and has again been expanded in both number of samples and represented platforms. Additionally, we systematically included suitable data from three more resources: ArrayExpress (15), cBioPortal (16) and TCGA(17) project. As in the previous database updates, we have also included data directly derived from publication supplements and from collaborative projects. Table 1 shows statistics of samples within the major sources. Table 2 reports the overall data growth and sample counts stratified by cancer loci since the last update (4).

**Table 1.** Statistics of samples from various data resources

Data source	GEO	ArrayExpress	cBioPortal	TCGA	Total
No. of studies	898	51	38	33	1939
No. of samples	63 568	4351	19 712	22 142	138 663
Tumor	52 090	3887	19 712	11 090	115 357
Normal	11 478	464	0	11 052	23 306
Classifications					
ICD-O (Topography)	100	54	88	157	209
ICD-O (Morphology)	246	908	265	140	491
NCIt	346	148	422	182	788
Collections					
Individuals	63 568	4351	19 712	10 995	127 549
Biosamples	63 568	4351	19 712	22 142	138 663
Callsets^a^	63 568	4351	19 712	22 376	138 930
Variants	5 514 126	118 4170	1 778 096	2 654 065	10 716 093

^a^set of variants from one genotyping experiment; ICD-O, International Classification of Diseases for Oncology; NCIt, National Cancer Institute Thesaurus.

**Table 2.** Data growth by cancer loci

Cancer loci	No.in 2014	No.in 2021
Hematopoietic and reticuloendothelial systems	5269	18 482
Lymph nodes	2345	5988
Breast	2271	15 790
Cerebellum	1439	3465
Brain, NOS	1342	6608
Cerebrum	1201	1712
Liver	1180	3237
Stomach	1155	3176
Skin	1073	3343
Connective, subcutaneous and other soft tissues	1058	2526
Kidney	1018	3617
Colon	1001	5182
Ovary	733	3963
Prostate gland	735	4485
Lung and bronchus	699	10 321
Nervous system, NOS	667	926
Urinary bladder	587	1961
Cervix uteri	529	1331
Peripheral nerves incl. autonomous	523	1479
Esophagus	454	1890
Pancreas	426	1620
Thyroid gland	404	1260
Heart, mediastinum and pleura	383	771
Bones, joints and articular cartilage	350	1205
Spleen	278	636
Other	4522	16 268
Total	31 642	115 359

The ‘ArrayExpress Archive of Functional Genomics Data’, hosted by EMBL-EBI, stores functional genomics data submitted by research groups and projects. In this update, we have incorporated the cancer-related genomic profiles which do not have corresponding GEO entries using our analysis pipeline. Overall, data from ArrayExpress added 3887 samples from 44 projects, which resolve to 143 distinct cancer types according to the National Cancer Institute Thesaurus (NCIt). Similar to the GEO data acquisition procedure, we have used a combination of text mining methods and expert curation for annotation of technical metadata and biomedical parameter.

The ‘cBioPortal for Cancer Genomics’ is an open-access resource for cancer genomics data, representing different types of molecular screening data from 19 712 samples, derived from 38 studies and mappable to 422 NCIt cancer types. The largest part of genomic data is based on WES analyses from the Memorial Sloan Kettering-Integrated Mutation Profiling of Actionable Cancer Targets or MSK-TARGET (18) pipeline, with CNA data accessed directly as segment files in genome version hg19/Genome Reference Consortium Human Build 37. Data were converted into Genome Reference Consortium Human Build 38 (GRCh38) with the ‘segment-liftover’ tool (19), and oncology classifications as well as relevant clinical data were incorporated into our database.

**Figure 1.**
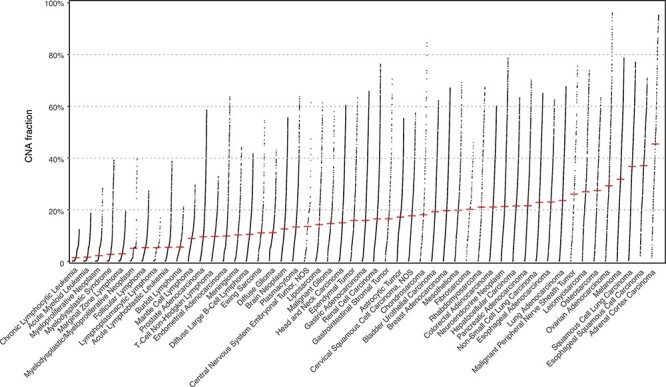
The currently available CNA data points in Progenetix and TCGA Progenetix database contain 115 357 cancer samples with 92 307 mapped to the 51 defined critical nodes in NCIt ontology tree and 23 050 samples not mapped to the tree (black), whereas TCGA repository contains 11 090 samples with 9103 samples mapped and 1987 samples not mapped to the tree (black). Colors of the stacked bar plot (left) match the branch colors on NCIt ontology tree (right).

TCGA project provides a set of multiomics data with extensive structured metadata annotation for a large collection of cancer types, currently through NCBI’s Genomic Data Commons Data Portal (https://portal.gdc.cancer.gov). In this update, we incorporated its copy number variation (CNV) profiling data as well as transformed the relevant clinical information into our system (Figure 1).

### Data processing update

Genomic profiling data in Progenetix originates from a large number of studies, which are based on different molecular-cytogenetics- and sequencing-based technologies. In order to maximize qualitative homogeneity of the final CNA calls, we prefer to download source files with the least amount of pre-processing and apply our in-house data processing pipeline from the arrayMap project (5). Currently, our analysis workflow handles the raw-data-based processing for 13 Affymetrix single nucleotide polymorphism (SNP) array platforms, including nine genome-wide arrays—10K (GPL2641), 50K (Hind240 and Xba240; GPL2004 and GPL2005), 250K (Nsp and Sty; GPL3718 and GPL3720), Genome-wide SNP (5.0 and 6.0; GPL6894 and GPL6801, respectively), CytoScan (750K and HD; GPL18637 and GPL16131) arrays (GPL-prefixed platform coding in brackets according to GEO standard)—as well as the four cancer-specific ‘Oncoscan’ arrays - GPL18602, GPL13270, GPL15793 and GPL21558 (accessible through GitHub repository *baudisgroup/a.m._process*). Our current model treats the most prevalent copy number as the baseline and derives the relative copy number gain and loss per sample based on the assumption that the relative gene dosage imbalance exerts pathophysiological effects in cancer biology.

### Allele-specific copy number variation

For the subset of SNP-array-based experiments—where the status of both alleles can be evaluated separately—we have analyzed allele-specific copy number data (ASCN) and incorporated 35 897 loss of heterozygosity (LOH) profiles into the database. ASCN potentiates new analysis on the same samples. First, probe-wise it gives an overview of germline variant landscape, as used in determining the ancestry background. Second, it allows detection of LOH events, including copy-number-neutral event (CN-LOH), which e.g. can be commonly observed in hematological malignancies due to a selective process for duplication of minor disease-prone germline alleles (20, 21). Lastly, it acts as a second reference for CNA to combat the variability caused by known wave artifacts from array technologies (22). For all SNP arrays, we have implemented a pipeline to determine probe-wise B-allele frequency (BAF) of SNP probes and perform subsequent segmentation (23, 24). Subsequently, we use ASCN to assess ancestry provenance of the samples (25) and store the LOH regions of the samples in our genomic variants database.

## Metadata updates

### NCIt ontology mapping

**Figure 4.**
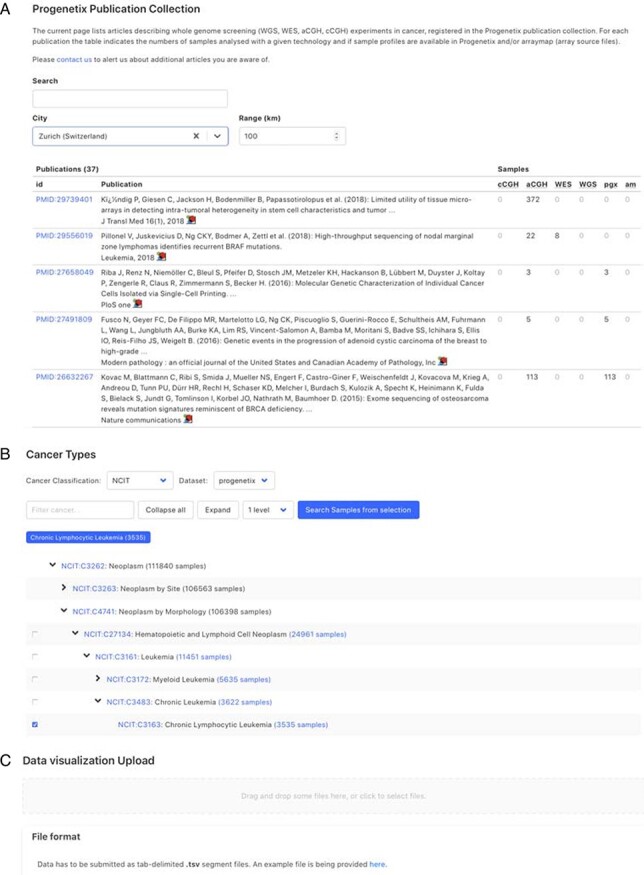
Demonstration of further functionality pages: A. Publication search; B. NCIt hierarchical tree navigation A: Cancer-genomics-associated publications are recorded with number of samples stratified by technology used. The publications can be filtered by keywords; B: Part of the sample subsets contained in Progenetix under the hierarchical NCIt classification tree. It allows for selection of sample subsets at different levels; C: User can upload custom segment files for data visualization.

Since its establishment, Progenetix has made use of the ‘International Classification of Diseases in Oncology’, 3rd Edition (ICD-O-3) (26) for cancer sample classification. While the combination of the ICD-O Morphology and Topography coding systems depicts diagnostic entities with high specificity, the current ICD-O is limited in its representation of hierarchical concepts and does not easily translate to modern ontologies. In comparison, NCIt (access through http://bioportal.bioontology.org/ontologies/NCIT) is a dynamically developed hierarchical ontology, which empowers layered data aggregation and transfer between classification systems and resources. However, due to the comparatively recent development and ongoing expansions, NCIt terms are rarely used in primary sample annotations. In the recent Progenetix update, we performed a data-driven generation of ICD-O—NCIt mappings and added the derived NCIt codes to all (existing and new) samples (mapping available through GitHub repository ‘progenetix/ICDOntologies’; manuscript in preparation), to take advantage of NCIt’s hierarchical structure for data retrieval, analysis and exchange (Figure 4B).

### Data summary based on the NCIt hierarchy tree

All cancer samples in Progenetix have been annotated with an NCIt code, resulting in currently 788 distinct NCIt terms. However, as the definition of increasingly specific NCIt terms outruns their incorporation into the hierarchical tree, so far 98 of these terms are not represented in the tree hierarchy. For better illustration, we define 51 prominent nodes under which we summarize and visualize the data collection (see Supplementary material for the selection procedure). This brings about additional 324 (60 in TCGA) terms not mappable to the selected nodes, resulting in 23 050 (1987 for TCGA) samples excluded from the summary tree counts (black bar in left panel of Figure 1). For terms with multiple occurrences in the tree, we define the preferred path to the selected node by prioritizing morphology-based separation. The sample collection in Progenetix compared to TCGA is summarized with reference to the NCIt coding system (Figure 1; Supplementary Table S1).

### CNV data content by cancer type

**Figure 2.**
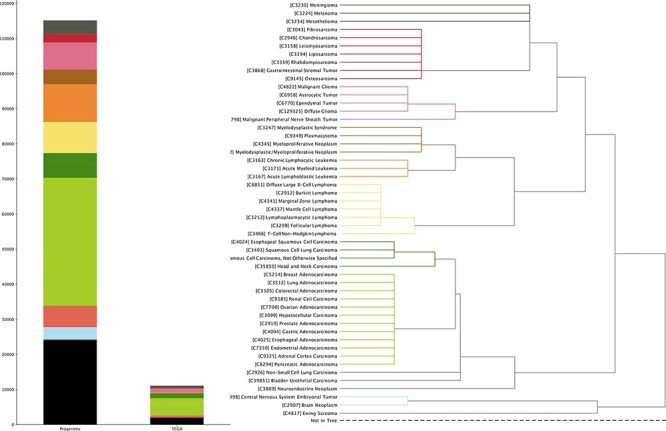
The genomic CNV fraction across 51 NCIt umbrella nodes Each dot represents one sample’s CNV fraction range from 0 to 1 and the red horizontal line indicates median CNV of the respective cancer type. Each cancer type contains between 104 and 11 804 CNV profiles (median 904; See Supplementary Table S1).

With cancer genomes grouped in the 51 NCIt nodes, we assessed their differences in the CNV landscape. The fraction of genome with a copy number alteration (CNV fraction) varies widely among the cancer types with a global median of 0.121 (Figure 2; Supplementary Figure S1). Among the most studied cancer types, breast carcinoma shows a consistent CNV profile as an earlier analysis with frequent chr1q, 8q, 16p, 17q, 20 gain and 8p, 16q, 17p, 18, 22q loss (27); the CNV patterns in cervical (chr3 gain) and colorectal (chr7, 8q, 13, and 20q gain and 8p, 17p, and 18 loss) carcinoma also correspond with previous observation (28), similar to T-cell non-Hodgkin lymphoma (29), myelodysplastic syndrome (30) and a number of malignant epithelial tumors (31). In addition, we also present the genome-wide LOH profile in the evaluated NCIt nodes clustered by their LOH landscape (average LOH profiles of 42 out of 51 with at least 20 samples are shown in Supplementary Figure S2; (32)). LOH profile of a cancer genome complements its CNV profile with the information of allelic loss. Here we highlight a few prominent patterns, which have been previously reported: chr3p and 9 in esophageal squamous cell carcinoma (33, 34); chr18q in colorectal carcinoma (35); and chr13q, 16q and 17p in hepatocellular carcinoma (36).

### Uberon anatomy ontology

While the ICD-O topography system provides organ- and substructure-specific mapping rooted in traditional clinical and diagnostic aspects of a ‘tumor entity’, ‘UBERON’ is a cross-species anatomical structural ontology system closely aligned with developmental processes (37). Its relationship structure allows integrative queries linking multiple databases (e.g. Gene Ontology (38) and Protein Ontology (39)) and description logic query within the same organism (linking related organs) and between model animals and humans. In this resource update, we have mapped all existing ICD-O T codes to ‘UBERON’ terms and additionally provided those as part of the ‘Monarch’ initiative (40), with our latest mapping table (made available through a GitHub repository ‘progenetix/icdot2uberon’).

### Provenance by geography

As part of the curated metadata provided in the sample representation, we have included geographic point coordinates for each individual sample. As this information is often missing from individual sample annotations, we have previously applied a mapping procedure to assign the samples’ approximate geographic origins (41, 42). For samples with the submitter’s contact available from repository entries, a default point location in the corresponding city was used—otherwise that of the corresponding author of the associated publication was used. Associated publications were also explored for more detailed descriptions of sample origin. Point coordinates for each city were obtained using the external geographic database GeoNames (www.geonames.org), as detailed previously.

### Provenance by ancestry group

While providing a good approximation for the geographic origin of cancer profiling data, which can e.g. be useful for epistemic validation and decision processes, the geographic location of the studies provides limited specificity regarding individual sample provenance, especially when assessing correlations between genomic variants and ancestral population background. Beyond the scope of high-penetrance variants like mutations in the BRCA1/2 (43, 44) or RetinoBlastoma (RB) genes (45) in cancer predisposition, other studies have asserted an influence of genetic background on tumor development (46–49). Previously we have developed a method for deriving ancestry groups from unmasked germline variants in cancer genomes, based on reference populations studied in the 1000 Genomes Project (25). For samples in Progenetix with accessible SNP data, population groups were assigned based on the reference categories mapped to Human Ancestry Ontology terms (Supplementary Table S2). Where available, the respective data are now represented under the ‘populations provenance’ schema for the corresponding biosample entries.

## Updated data access modalities

Since the last release, we have adopted the GA4GH data schema standards and migrated to Phenopackets (50)-formatted response delivery with modified data access points in the user interface. Information about API methods are provided through the documentation pages (https://info.progenetix.org/categories/API).

### Data standards

In many genomic repositories, databases are structured around experimental outcomes (e.g. variants from a DNA sequencing experiments as collections of VCF files). Recent attempts in evaluating sensible meta-schemas for the representation of genomic variants and related biological or technical metadata, especially with respect to empowering data federation over flexible, networked resources, have led to a set of emerging meta-models and data schemas (51). The data storage and representation models for the Progenetix resource have been designed to comply with concepts developed by the previous GA4GH Data Working Group (12, 52) and subsequent GA4GH work streams, documented e.g. by the ‘SchemaBlocks’ initiative (http://schemablocks.org). One of the core concepts is the ‘individual—biosample(s) - variants’ meta-model, which is applicable to cancer-related analyses with potentially multiple samples representing different stages in the course of disease as well as the underlying genomic background. This hierarchical model provides a solid representation and connection between the physical source of the data and the logical genotyping information and adapts to various scenarios for data aggregation and analysis.

### User interface

The completely re-designed user interface provides flexibility and versatility in query parameters and types and optimized the response delivery. Technically, the query interface for retrieval of sample specific data is built on top of a forward-looking implementation of the GA4GH Beacon API (13) with features from the upcoming version 2 of this standard.

**Figure 3.**
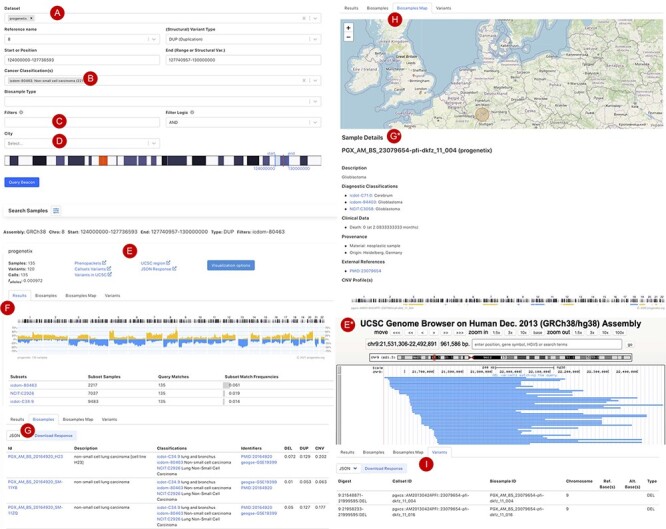
Beacon-style query using fuzzy ranges to identify biosamples with variants matching the CNA range This example queries for a continuous, focal duplication covering the complete MYC gene’s coding region with < = 6 Mb in size. A: Filter for dataset; B: filter for cancer classification (NCIt and ICD-O-3 ontology terms available); C: additional filter, e.g. Cellosaurus; D: additional filter for geographic location; E: external link to UCSC browser to view the alignment of matched variants; F: cancer type classification sorted by frequency of the matched biosamples present in the subset; G: list of matched biosamples with description, statistics and reference. More detailed biosample information can be viewed through ‘id’ link to the sample detail page; H: matched variants with reference to biosamples can be downloaded in json or csv format.

Figure 3 shows the current web interface to perform a CNA query with start and end position range with filter options for cancer type, tissue location, morphology, cell line or geographic location. The top panel of the result page shows a summary with the number of matched samples, variants, calls and the frequency of alleles containing the CNA (Figure 3E). The ‘Phenopackets’ link returns a json document of biosamples with the phenopacket-formatted response. The ‘UCSC region’ links externally to a University of California Santa Cruz (UCSC) browser track providing an overview of the genomic elements which map to the region of the observed variants. Also, customized visualization is enabled in the linked page ‘visualization options’, e.g. for selected chromosomal regions and grouping by subsets or studies. The lower panel is organized in four sections: (i) the ‘Result’ tab (Figure 3F) shows the genome-wide CNA by the percentage of samples with yellow (+) as CN gain and blue (−) as CN loss. Below the CNA plot is a table showing the list of subsets as defined by ICD-O-3 and NCIt Ontology terms sorted by frequency of matched samples within that subset. (ii) the ‘Biosamples’ tab (Figure 3G) shows information of matched biosamples, i.e. description, classifications and external identifiers. The table can be downloaded in json or csv format. The further detail of the biosample can be accessed by clicking the biosample id. (iii) The ‘Biosamples Map’ tab (Figure 3H) shows a world map with the matched geological locations highlighted. (iv) the ‘Variants’ tab (Figure 3I) shows the variant ‘digest’ (concatenated format with chromosome, start and end position, and type of the CNA) and its corresponding biosample and callset. Likewise, the table can be downloaded in json or csv format.

Figure 4 shows the additional functional interfaces and services provided by the Progenetix project. Users can search for publications or studies by publication title, author names or the geographic location of the research center. Then, navigation extends to the summary of publications with the number of samples catalogued by technology and availability in database as well as options to visualize the associated samples (Figure 4A). Users can also access samples from the NCIt hierarchical tree or other classification systems (e.g. ICD-O and UBERON) to select a subset of cancer types for summary statistics and visualization (Figure 4B). Alternatively, users can also upload their own data for single or multiple samples to visualize genome-wide CNA (Figure 4C). In addition, a list of studies and cohorts can be selected in the navigation menu, including arrayMap (probe-specific arrays from published studies (5)), diffuse intrinsic pontine glioma cohort (53) and the ‘cancer signature’ cohort (54). All the functionalities and provided services are detailed in the documentation pages at info.progenetix.org, which invite request submission through the GitHub ‘issues’ tracker.

## Other improvements

### Genome version update

All samples have been updated to GRCh38. The process has been completed in a step-wise manner. Preferably, for samples with available probe-specific array data, either GRCh38 mapped platform data files were used for re-processing of the original files or alternatively, a lift-over of the probe data and subsequent re-segmentation was performed. For those cases where only called CNA data had been collected, we applied our recently published ‘segment-liftover’ tool (19) for the efficient re-mapping of continuous segments. Overall, more than 99.99% of probes and more than 99% of segments could be recovered successfully.

### Cell line collection

Cancer cell lines are important models for understanding the molecular mechanisms of malignant diseases and have a prominent role in pharmacological screening procedures. Besides the primary tumor data, the Progenetix data collection also includes genomic profiling experiments using *in vitro* models. Recently, we introduced a systematic update of cell line annotations based on ‘Cellosaurus’, a comprehensive knowledge resource on cell line data with extensive annotations and mappings to a variety of classifications and ontologies (55). We meticulously assigned Cellosaurus ids for the cancer cell line samples as well as the ICD-O morphology and topography codes based on the NCIt term annotated by Cellosaurus. At this time, Progenetix includes a total of 5764 samples corresponding to 2162 different cancer cell lines, representing 259 different cancer types (NCIt). While so far we provide the option to search for cell lines by applying a ‘cellosaurus’ filter either in the web interface (e.g.‘cellosaurus: CVCL_0030’ for ‘HeLa’ cell line samples) or in the API query, work on a dedicated cell line data access tool is underway.

## Conclusion

The Progenetix resource provides an extensive collection of oncogenomic data with a focus on individual genome-wide CNA profiles and the use of modern ontologies and data schemas to render curated biological and technical metadata, as well as thorough references to external repositories and annotation resources. Through aggregation of data from thousands of individual research studies as well as several consortium-derived collections, to our knowledge Progenetix database currently constitutes the largest public, freely accessible resource for pre-computed CNA profiles and associated phenotypic information and additional metadata dedicated to cancer studies. While the application of uniform genomic data formats and a benchmarked data processing pipeline minimizes biases from separate studies, the forward-looking implementation of emerging ontology standards facilitates the integrative and comparative analysis across a vast range of cancer types. The tight integration with GA4GH product development and standardization processes guarantees the compatibility with emerging data federation approaches and the widest re-utilization of the resource’s data. For the future, besides the continuous maintenance and expansion of the existing data types, we will work toward enhancing clinical and diagnostic annotation, expanding cross-database references and the types of genomic variant data as well as active data sharing and integration through networked services and platforms.

## Supplementary Material

baab043_Supp
