# Supplementary material for: The Progenetix oncogenomic resource in 2021
Source: Database (Oxford). 2021 Jul 17;2021:baab043. doi: 10.1093/database/baab043 (PMC8285936; doi:10.1093/database/baab043)
Supplement: baab043_Supp [file baab043_supp.zip › supplementary_material-new.docx]

**Supplementary material**

**Material and Methods**

Selection of 51 NCIt types

The procedure to derive the 51 NCIt types is done by iterating the following steps until no updates:

1. hierarchical tree back-tracing (programmatic)
2. supervised selection based on the cancer type/biology
3. (Re-do step 1with new information from step 2)

The first step takes the current NCIt term for every sample in the database, assigns them to the parent term, and counts the number of samples in the derived parent terms. The second step manually evaluates the parent terms and replaces the current term unless: a) the parent term is too broad and contain heterogeneous types (e.g. Adenocarcinoma); b) there are more than 2000 samples in the parent term. For some specific terms, there are multiple lineages (multiple parent terms), the parent term is selected to prioritize the functional (morphology) lineage instead of the location (site) lineage.

Through this procedure, the unique 788 most accurate NCIt terms in Progenetix are reduced to 51 “umbrella terms”, with sample counts ranging from 104 (lymphoplasmacytic lymphoma) to 11762 (breast adenocarcinoma).

**Appendix**

*Modified features compared to last published version*

We no longer offer permanent data storage for users due to data privacy regulations (i.e. GDPR adherence, storage of potentially sensitive data not under our control).

However, based on a number of recurring requests, we provide users an upload with a dedicated “Upload & Plot” function for genomic segmentation data, through which users can visualize their own CNV data with the Progenetix plotting options for individual samples as well as multiple sample histograms. For that purpose we provide a simple “.pgxseg” file format which users can adopt to fully utlize the plotting libraries, e.g. grouping and labels.

As a public resource, Progenetix develops in the direction to answer the needs of the scientific community. We have a dedicated GitHub repository for documentation ([info.progenetix.org](http://info.progenetix.org)) at “[progenetix](https://github.com/progenetix)/[progenetix.github.io](https://github.com/progenetix/progenetix.github.io)", where users are encouraged to submit suggestions and requests as new issues.
