# Supplementary figures and images for: The Progenetix oncogenomic resource in 2021
Source: Database (Oxford). 2021 Jul 17;2021:baab043. doi: 10.1093/database/baab043 (PMC8285936; doi:10.1093/database/baab043)

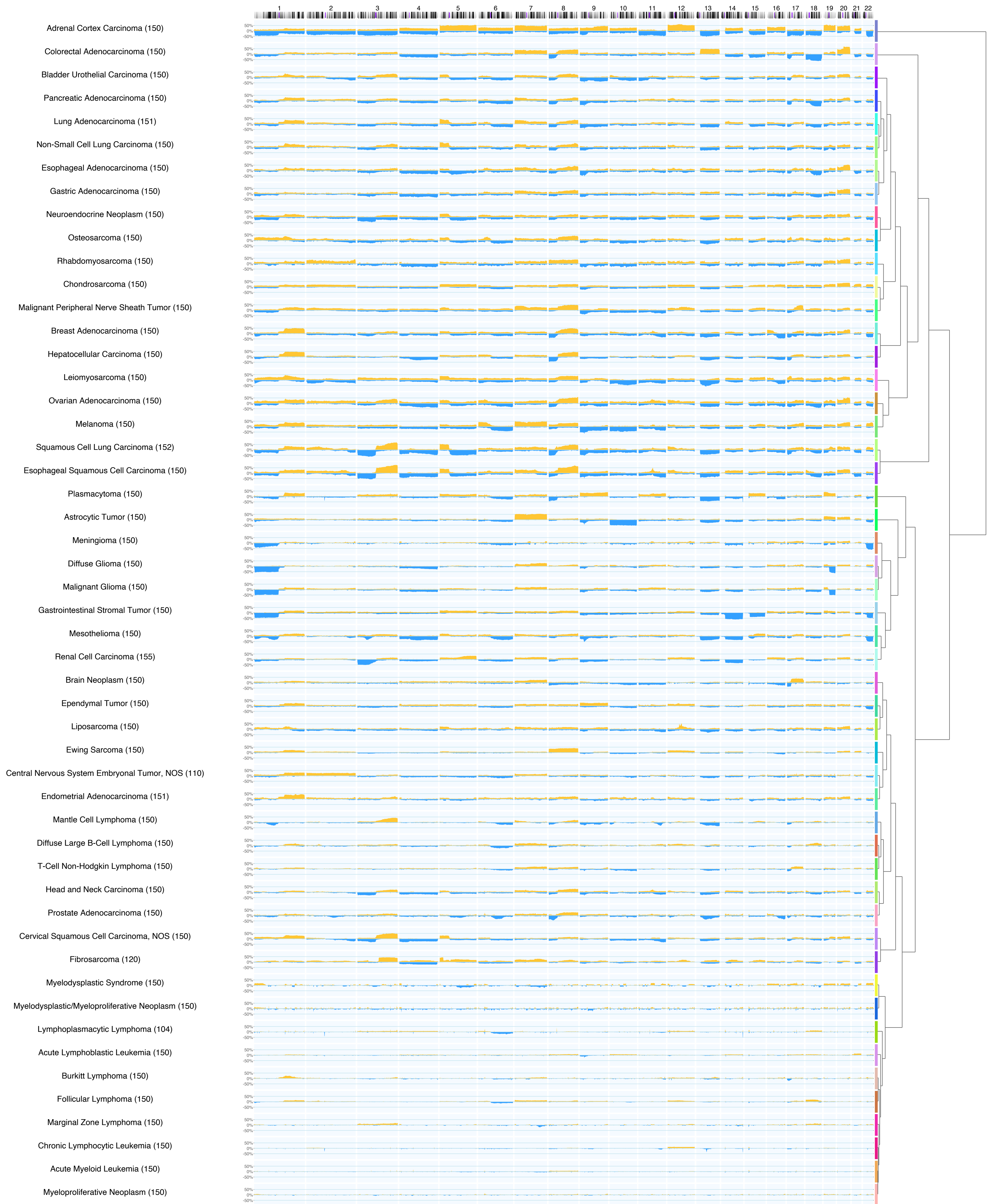

Supplement: baab043_Supp [file baab043_supp.zip › SuppFig1_51_NCIt_CNV_landscape.pdf]

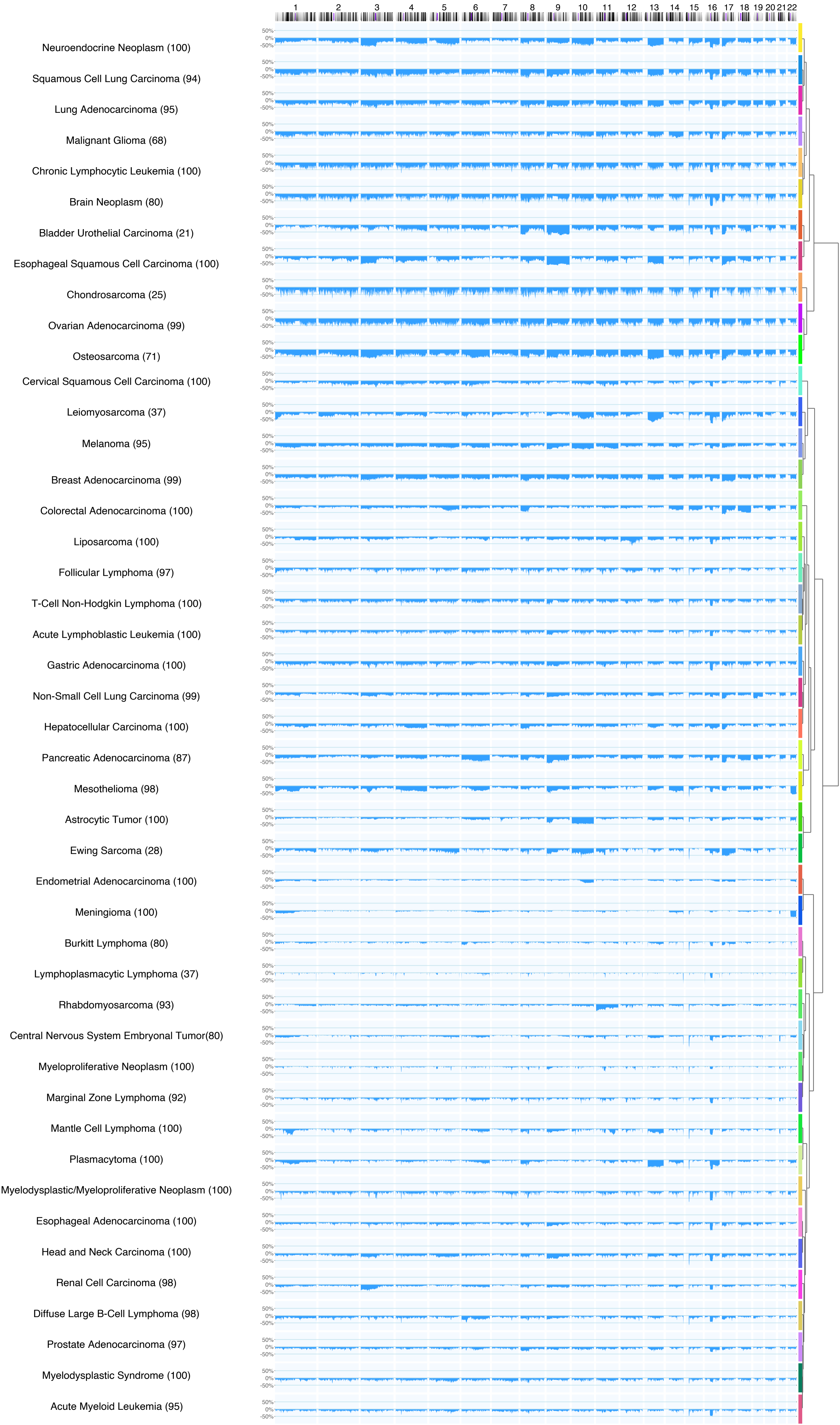

Supplement: baab043_Supp [file baab043_supp.zip › SuppFig2_51_NCIt_LOH_landscape.pdf]
